# Gray Matter Characteristics in Mid and Old Aged Adults with ASD

**DOI:** 10.1007/s10803-016-2810-9

**Published:** 2016-05-13

**Authors:** P. Cédric M. P. Koolschijn, Hilde M. Geurts

**Affiliations:** Dutch Autism & ADHD Research Center, Brain and Cognition, University of Amsterdam, PO Box 15915, 1001 NK Amsterdam, The Netherlands; Amsterdam Brain and Cognition (ABC), University of Amsterdam, Amsterdam, The Netherlands; Dr. Leo Kannerhuis, Amsterdam, The Netherlands

**Keywords:** Autism, Gray matter volume, Cortical thickness, Surface area, Gyrification, Adults

## Abstract

It is widely acknowledged that the brain anatomy of children and adolescents with autism spectrum disorder (ASD) shows a different developmental pattern then typical age-matched peers. There is however, a paucity of studies examining gray matter in mid and late adulthood in ASD. In this cross-sectional neuroimaging study, we, performed vertex-wise whole-brain and region-of-interest analyses of cortical volume, thickness, surface area, and gyrification index in 51 adults with and 49 without ASD, between 30 and 75 years. There was significant age-related volume loss and cortical thinning, but there were no group differences. The lack of significant anatomical differences between intellectual able individuals with and without ASD, suggests that ASD is not (strongly) related to gray matter morphology in mid and late adulthood.

**Electronic supplementary material:**

The online version of this article (doi:10.1007/s10803-016-2810-9) contains supplementary material, which is available to authorized users.

## Introduction

Autism spectrum disorder (ASD) is a tale of many brain regions, as the anatomical signature of ASD seems to be marked by age-specific changes. ASD is a lifelong disorder associated with early neurodevelopmental deficits, with one of the most consistent findings being accelerated brain growth during early childhood (Courchesne et al. 2007) and deceleration during late childhood and adolescence (Courchesne et al. 2011; Duerden et al. 2012; Stanfield et al. 2008). The findings predominantly point toward anomalies (but see Haar et al. 2014) in the frontal and parietal lobes, the limbic system, and the cerebellum, though directions and magnitude vary across studies (Cauda et al. 2011; Duerden et al. 2012; Nickl-Jockschat et al. 2012; Stanfield et al. 2008; Via et al. 2011). This is further substantiated by post-mortem research contributions of different cellular factors (e.g. neuronal numbers, dendritic growth and number of synapses, and number and size of glial cells) in frontal, parietal and anterior cingulate cortex, amygdala and the cerebellum (Schumann and Nordahl 2011). For adults with ASD it is difficult to pinpoint specific affected brain regions, because the majority of studies have age-ranges including adolescence and research on typical brain development has shown protracted brain maturation in adolescence (Giedd et al. 2009; Koolschijn and Crone 2013; Koolschijn et al. 2014; Ostby et al. 2009). Given the aberrant neurodevelopmental course of ASD, inclusion of children and adolescents hampers interpretation of adult-specific abnormalities. Hence, what happens to the brain of people with ASD when they age is unknown as most research focused largely on ASD in children, adolescents, and young adults (Howlin and Moss 2012; Mukaetova-Ladinska et al. 2012). Therefore, we will focus on middle and older aged adults with ASD.

The aforementioned brain volumes have been studied successfully, and extensively (Cauda et al. 2011; Duerden et al. 2012; Nickl-Jockschat et al. 2012; Stanfield et al. 2008; Via et al. 2011) and these studies have been highly informative. However, volumetry is a composite measure of anatomical properties such as thickness, surface area, and gyrification. These properties are under diverse genetic control and have distinct trajectories in the typical developing brain (Panizzon et al. 2009; Raznahan et al. 2011; Winkler et al. 2010). For example, the radial unit hypothesis of cortical development suggests that cortical thickness is influenced by the number or the size of cells within a column at least some brain regions, while the cortical surface area is influenced by the number and spacing of cortical columns (Rakic 1988, 2000). With respect to gyrification, the process by which the cortical surface morphology is altered to create the sulcal and gyral regions, it is thought that it reflects both interregional connectivity and optimal intracortical organization (Van Essen 1997; White et al. 2010). For ASD, examination of cortical gyrification is potentially informative about pathological deviations in neurodevelopment, as abnormalities in interregional connectivity have been reported in children and adolescents and young adults with ASD (Vissers et al. 2012). Hence, in the current study we will, next to volume, focus on each of these three anatomical properties of gray matter.

When focusing on studies examining cortical surface measures like thickness, surface area, and gyrification patterns in people with ASD, only a few studies also included adults with ASD and we see rather mixed results. For instance, with respect to cortical thickness the temporal cortex has been reported thicker (Scheel et al. 2011), thinner (Hadjikhani et al. 2006; Raznahan et al. 2010; Wallace et al. 2010), or not different from a comparison group (COM) (Zielinski et al. 2014). In a small adult sample, cortical thinning was observed in various brain areas including, prefrontal, orbito-frontal, and anterior cingulate cortex (Hadjikhani et al. 2006). Yet other studies reported a scattered pattern of both cortical thickness increases (frontal and temporal regions; (Ecker et al. 2013)), and decreases (occipital and temporal regions) (Wallace et al. 2010) compared to a comparison group. Moreover, also larger (frontal and orbito-frontal regions, temporal and motor areas), and lower (primarily parietal regions) surface area was found in these adult ASD males (but see Wallace et al. 2010). In a large longitudinal study with children and young adults, abnormal age-related cortical thickness trajectories were found and emerged in occipital, frontal and parietal regions (Zielinski et al. 2014). A few studies have examined gyrification patterns in ASD in mixed adolescent/adult samples. These studies reported higher gyrification indices in ASD in bilateral posterior regions (Wallace et al. 2013) and in the left supramarginal gyrus as a function of age (Libero et al. 2014) compared to individuals without ASD. Furthermore, higher gyrification indices of the left frontal cortex have been reported in children and adolescents with ASD, but not in adults with ASD suggesting normalization in adults (Hardan et al. 2004). Taken together, although previous studies have examined various morphological properties in adults with ASD, no studies to date have explicitly performed a concurrent comprehensive evaluation of (sub)cortical structure characteristics in middle and old aged adults with ASD. Therefore, we tested individuals with and without ASD between 30 and 75 years, with a mean age around 50 years. Given that in most adult gray matter studies the maximum age is around 50 years, the inclusion of older adults in the current study gives us the unique opportunity to explore whether in ASD we will observe the similar age-related declines as in typical aging (Raz et al. 2010; Walhovd et al. 2005).

Besides whole brain vertex-based analyses (Raznahan et al. 2010; Zielinski et al. 2014), we extracted the four morphological properties (cortical thickness, volume, surface area and *l*GI) from the lobes (Raznahan et al. 2010), and gray matter (GM) volumes on the subcortical level for replication purposes (Osipowicz et al. 2015; Raznahan et al. 2010; Zielinski et al. 2014). Even though findings are mixed in the adult ASD literature, we hypothesized abnormalities in cortical volume and thickness, but not surface area, in both frontal and temporal regions in middle and older aged adults with ASD compared to our comparison group (Ecker et al. 2013; Raznahan et al. 2010). Moreover, we expected aberrant gyrification in temporal-parietal regions, but the specific direction of these abnormalities could be either way (Hardan et al. 2004; Libero et al. 2014; Schaer et al. 2013; Wallace et al. 2013). Subcortically, we expected primarily abnormalities in the hippocampus, amygdala, and caudate nucleus (Cauda et al. 2011; Duerden et al. 2012; Nickl-Jockschat et al. 2012; Stanfield et al. 2008; Via et al. 2011). However, each of these hypotheses were based on studies including younger adults as compared to the current study. It could well be that after the aberrant neurodevelopmental trajectories in early life, the brains of ASD individuals tend to rearrange towards a “normal” shape as two recent longitudinal studies demonstrated pseudonormalization in most brain regions during adolescence and young adulthood (Zielinski et al. 2014), and atypical volume decline (Lange et al. 2015). Based on these findings we would expect a general converging pattern showing fewer differences between groups with increasing age (Raznahan et al. 2010). Therefore, we also explored whether age-related differences in ASD are parallel, convergent or divergent relative to our comparison group.

## Methods

### Participants

A total of 51 individuals with and 49 without ASD between 30 and 75 years were recruited from a cohort of participants of a large-scale behavioral study “Autism & Aging: A double jeopardy?” conducted at the Dutch Autism & ADHD Research Center, Amsterdam, The Netherlands. Participants were recruited via advertisements, our website and personal contacts, and from mental health clinics specialized in the assessment and care of adults with ASD (Lever and Geurts 2015; Lever et al. 2015). All individuals with ASD received their clinical ASD diagnosis by a multidisciplinary team with clinicians experienced in the assessment of ASD. Hence, we are following the Dutch Multidisciplinary Guidelines for ASD assessment in adults (Kan et al. 2013) and the UK NICE guidelines for identification and assessment of ASD in adults (http://www.nice.org.uk/guidance/CG142). To verify the clinical diagnosis we used the following diagnostic inclusion criteria for ASD participants: (1) a formal diagnosis of ASD prior to inclusion; (2) confirmation of diagnosis on the Autism Diagnostic Observation Schedule module 4 (Lord et al. 1989)) and/or Autism-Spectrum quotient, 50-item list (Baron-Cohen et al. 2001): 33 individuals had a score above the cutoff of the ADOS (≥7) and those not scoring above this cut off did score above the AQ cutoff (≥26); for similar approaches see (Ecker et al. 2012; Lai et al. 2013). Please note that, although the evaluation of childhood problems was part of the clinical assessment procedures, obviously, with the age-range of our participants an interview such as the Autism Diagnostic Interview-Revised (ADI-R; (Lord et al. 1994)) is not feasible or reliable.

Secondary inclusion criteria included an estimated IQ above 80 which was based on two subtests of the WAIS-IV (Wechsler 1981), and an absence of MRI-contraindications. Furthermore, participants were not selected for the MRI-study in case of a self-reported history of neurological disorders, chronic somatic illnesses, learning disabilities or schizophrenia based on the Mini-International Neuropsychiatric Interview (Sheehan et al. 1998). For the comparison group, an additional exclusion criterion was a first or second-degree family member with ASD. This was based on self-report. There were no between group differences for IQ, age, sex, and handedness (Table 1). All participants gave written informed consent for the study and received fixed payment for participation and travel reimbursement. Of note, all individuals selected for the MRI-study had been screened in the initial cohort study for capacity to consent based on the mini–mental state examination and IQ estimates. The internal review board from the University of Amsterdam approved the study (#2013-PN-2668).

**Table 1:** Demographic variables

Description	ASD^a^	COM	Statistics ASD vs COM
	N = 51	N = 49	
#Males (%)	35 (69 %)	32 (65 %)	*χ* ^*2*^ = .13, *p* = .724
Age (SD)[range]	51.46 (12.61) [30.04–73.98]	50.14 (11.94) [30.62–73.77]	*F* = .29, *p* = .592
IQ (SD) [range]	116.31 (16.21) [86–155]	111.59 (15.78) [80–141]	*F* = 2.18, *p* = .143
MMSE total score (SD) [range]	29.18 (.95) [27–30]	28.98 (1.11) [26–30]	*F* = .91, *p* = .343
Level of Educational Attainment^b^	1/16/22/12	1/11/27/10	*χ* ^*2*^ = 1.58, *p* = .664
Handedness			*χ* ^*2*^ = .083, *p* = .959
Left/Right/Ambidexter	5/43/3	4/42/3	
Age first diagnosis	45.89 (13.84) [11.22–68.08]	*N.A.*	
ADOS Total^h^	7.94 (3.31)	*N.A.*	
Language & Communication	2.57 (1.35)	*N.A.*	
Social reciprocity	5.37 (2.47)	*N.A.*	
Fantasy	1.12 (.52)	*N.A.*	
Restricted & Repetitive Behaviors	.29 (.58)	*N.A.*	
ADOS cutoff (< 7)^c^	18 (35 %)	*N.A.*	
AQ Total	36 (6.64)[19–47]	12.98 (5.89)[4–26]	***F*** **=** **349.98,** ***p*** **<** **.001**
AQ-cutoff (< 26 ASD, > 23 COM)	4 (8 %)	0	
Medication N (%)	40 (78 %)	19 (39 %)	***χ*** ^***2***^ **=** **16.25,** ***p*** **<** **.001**
Antidepressants	15 (29 %)	2 (4 %)^d^	***χ*** ^***2***^ **=** **11.36,** ***p*** **=** **.001**
Antipsychotics	8 (16 %)	0	***χ*** ^***2***^ **=** **8.36,** ***p*** **=** **.004**
Sedatives	7 (14 %)	0	***χ*** ^***2***^ **=** **7.23,** ***p*** **=** **.007**
Stimulants	6 (12 %)	0	***χ*** ^***2***^ **=** **6.13,** ***p*** **=** **.013**
Antiepileptics^e^	2 (4 %)	1 (2 %)	*χ* ^*2*^ = .30, *p* = .582
Antiparkinson^f^	1 (2 %)	0	*χ* ^*2*^ = .97, *p* = .325
Migraine	4 (8 %)	0	***χ*** ^***2***^ **=** **4.00,** ***p*** **=** **.045**
Non-psychotropic medication^g^	26 (51 %)	17 (35 %)	*χ* ^*2*^ = 2.70, *p* = .100

Numbers in bold reflect significant between group differences

*ASD* autism spectrum disorder, *COM* comparison group, *ADOS* Autism Diagnostic Observation Schedule, *AQ* Autism-Spectrum quotient, *MMSE* Mini-mental state examination, *N.A.* not applicable

^a^For the ADOS-only group (above cutoff > 7) there were no differences in demographics compared to COMs: N = 33 (23Males), mean age: 48.77 (11.50) [33.04–70.84], all *p*’s > .09, except for AQ-score F = 277.12, *p* < .001

^b^The numbers between the slashes indicate the number of participants who had pre-vocational education/junior general secondary or vocation education/senior general secondary education or vocation colleges/university education based on the Verhage scale (Verhage 1964)

^c^All subjects below threshold scores on the ADOS, had scores *above* the clinical cut-off for the AQ

^d^One case for mild depressive complaints (no formal diagnosis), the other for nerve damage

^e^Anticonvulsant class medication use was prescribed for non-epileptic related problems

^f^Prescribed for non-Parkinson-related problems

^g^Includes a.o.: blood pressure/thinner, antihistamines, cholesterol, sleeplessness, asthma, heartburn, diabetes

### Data Acquisition

All participants were scanned on a 3-Tesla whole body Philips Achieva MRI system with a 32-channel head coil (Best, The Netherlands). Two high-resolution T1-weighted anatomical scan were obtained: 3D-T1-weighted scan: TR = 8.2 ms; TE = 3.8 ms, 220 slices, voxel-size = 1 mm^3^, FOV = 240 × 188, matrix = 240, 2D SENSE directions: P(Right-Left) = 2.5, S(Food-Head) = 2).

### Image Analysis

Cortical reconstruction and volumetric segmentation was measured automatically using FreeSurfer5.3 (http://surfer.nmr.mgh.harvard.edu/ (Dale et al. 1999; Fischl and Dale 2000). Details of the surface-based cortical reconstruction and subcortical volumetric segmentation procedures have been extensively documented previously (Dale et al. 1999; Fischl and Dale 2000; Fischl et al. 2004; Segonne et al. 2004). In short, the FreeSurfer pipeline performs motion correction on the T1-images, automatically removes non-brain tissues (Segonne et al. 2004), transforms volumetric data to a common atlas, performs intensity normalization, topology correction (Fischl et al. 2004; Segonne et al. 2007) and defines the boundaries of the gray/white matter and pial surface (Dale et al. 1999; Fischl and Dale 2000). The volumetric subcortical segmentation procedure automatically classifies brain tissue into multiple distinct structures such as cerebral and cerebellar gray and white matter, and subcortical structures (Fischl et al. 2002, 2004). For the purposes of the current study, automated image surfaces and segmentations were inspected and screened for quality control but were not manually edited, in order to maintain the objectivity of results. No large deformities such as failure to segment were found in this study. Lobar segmentations were automatically computed by FreeSurfer using the “mri_annotation2label with –lobesStrict”-command. Intracranial volume (ICV) was determined by a validated automated method known to be equivalent to manual intracranial volume estimation (Buckner et al. 2004).

### Local Gyrification Index

Local gyrification indices (*l*GI) were obtained with the method developed by Schaer implemented in FreeSurfer (Schaer et al. 2008, 2009). The *l*GI is measured at each vertex using previously validated algorithms (Schaer et al. 2008). *l*GI is a surface-based measure of the degree of cortical folding that quantifies the amount of cortex buried within the sulcal folds in the surrounding circular region. Thus *l*GI is a ratio of the total cortical surface area to a reference surface, with higher indices implying a greater degree of gyrification. Details of the processing pipeline are fully described elsewhere [https://surfer.nmr.mgh.harvard.edu/fswiki/LGI, (Schaer et al. 2008, 2009, 2012)].

### Statistical Analyses

#### Cortical Thickness, Cortical Gray Matter Volume, and Surface Area

The cortical thickness, volume, and surface data were averaged across participants in the spherical coordinate system after smoothing FWHM 15 mm, and FWHM 5 mm for *I*GI measurements. Comparisons between individuals with and without ASD were performed using vertex-wise analyses using a general linear model approach in QDEC (Query, Design, Estimate, Contrast; statistical interface of FreeSurfer). The analyses included models per hemisphere for cortical volume, surface area (pial), cortical thickness and *I*GI. We compared whole-brain differences for all metrics with group (diagnosis), age and group-by-age interactions. Based on earlier reports of non-linear patterns of age-related GM changes (e.g. (Walhovd et al. 2005; Walhovd et al. 2011)) we also added age-squared (and group-by-age-squared interactions) as a predictor in our analyses. Models for GM volume were regressed out for ICV for all comparisons. In addition, sex and handedness were used as covariates in all analyses. Results of all GLM’s were corrected for multiple comparisons using a false discovery rate (FDR) of *p* < .05 (Genovese et al. 2002). In case of significant between-group results, Spearman’s correlations were computed for the relationship between the surface measures and AQ-scores (as the AQ-scores were not normally distributed in both groups [ASD: Kolmogorov–Smirnov = .137, *df* = 51, *p* = .018; COM: Kolmogorov–Smirnov = .176, *df* = 49, *p* = .001)].

The volumes of all (sub)cortical structures and ROIs were averaged across hemispheres within participants, except for the laterality analyses. Stepwise regression analyses were performed on the whole sample with ICV, then group, age, group-by-age interaction term, followed by sex and handedness as predictors. We corrected for multiple comparisons using a Holm-Bonferroni correction (Holm 1979). Taking a conservative approach, all between-group analyses were also performed with the ADOS-only group (i.e., those individuals with ADOS-scores above cutoff (> 7), see also note below Table 1). Findings are reported only for significant results unless otherwise specified.

### Lateralization Index

Based on earlier laterality findings in brain anatomy in ASD (e.g. (Ecker et al. 2010); laterality-indices (left–right/left + right); (Hadjulis et al. 2004)), which reflect the magnitude of left > right asymmetry of a measure, were computed for regional and lobar regions. The asymmetry index for each measure was subsequently compared between ASD and COM using GLMs with handedness [given the possible association with cerebral asymmetry (Corballis 2014)], but also age and sex as covariates.

## Results

### Whole Brain Vertex-Wise Analyses

No between group differences or group-by-age interactions were found for whole-brain vertex-wise analyses of GM volume, thickness, surface area or *l*GI after correction for multiple comparisons, and taking sex into account. Significant bilateral age-related volume loss and cortical thinning was found, but no age-related differences in surface area or *l*GI were found (Fig. S1). The results remained similar in a conservative exploratory approach including only people with ASD above ADOS cutoff (>7). Due to the skewed sex distribution and taking into account that most other studies are limited to males, we ran exploratory analyses taking sex into account and limiting our sample to males only. However, none of these sub-analyses revealed differences between groups on a whole brain level.

### Lobar and Subcortical Analyses

 Table 2 shows the results for the lobar analyses for each of the morphometric properties. Similar to the whole-brain vertex-wise analyses, no group or group-by-age interactions were found. Age-related effects were prominent for cortical thickness and volume, and frontal *l*GI, but not for surface area after correction for multiple comparisons. Age effects remained significant when considering the ADOS-only group (Table S2). As some may argue that raw volumes are more informative compared to ICV-corrected volumes, we also performed exploratory regression analyses without taking ICV into account. The results resemble our ICV-corrected findings.

**Table 2:** Lobar regression analyses for all morphometric measures

A	Volumes^a^					
	Lobes	Description	β	*p*	R^2^-model	*p*–F-change
	ACC	Age	−.369	**.001**	.541	**<.001**
	Frontal	Age	−.376	**<.001**	.618	**<.001**
	Insula	Age	−.116	.235	.586	**<.001**
	Occipital	Age	−.452	**<.001**	.589	**<.001**
	Parietal	Age	−.435	**<.001**	.73	**<.001**
	Temporal	Age	−.395	**<.001**	.53	**<.001**

B	Thickness					
	Lobes	Description	β	*p*	R^2^-model	*p*–F-change
	ACC	Age	−.615	**<.001**	.232	**<.001**
	Frontal	Age	−.512	**<.001**	.268	**<.001**
	Insula	Age	−.456	**<.001**	.125	**.005**
	Occipital	Age	−.663	**<.001**	.346	**<.001**
	Parietal	Age	−.721	**<.001**	.464	**<.001**
	Temporal	Age	−.51	**<.001**	.269	**<.001**

C	Surface area					
	Lobes				R^2^-model	*p*–F-change
	ACC				.028	.436
	Frontal				.027	.454
	Insula				.007	.869
	Occipital				.03	.398
	Parietal				.047	.29
	Temporal				.035	.333

D	*l*GI					
	Lobes	Description	β	*p*	R^2^-model	*p*–F-change
	ACC				.071	.07
	Frontal	Age	−.376	**.007**	.201	**.015**
		Sex	−.232	.015		
		Sex × age^a^	−.146	.62		
	Insula	Age	−.28	.041	.204	**.001**
		Sex	−.323	.001		
		Sex × age^a^	.66	.023		
	Occipital				.069	.076
	Parietal	Age	−.312	.024	.189	**.005**
		Sex	−.273	.005		
		Sex × age^a^	.291	.775		
	Temporal				.077	.051

Numbers in bold represent significant effects after Holm-Bonferroni correction

*ACC* anterior cingulate cortex, *lGI* local gyrification index

^a^With ICV correction

In Table 3 we report the results from the subcortical analyses. Similar to the lobar analyses, only age effects were found for the whole and ADOS-only group (Table S4), except for intracranial volume. Here we also report the regression results without taking ICV into account. The results resemble our ICV-corrected findings (right half of Table 3), and illustrate that significant sex differences, both lobar and subcortical, are all explained by ICV.

**Table 3:** Volumes of cortical and subcortical brain structures

Brain area	Description	β	*p*	R^2^-model	*p*–*F*-change model	Description	β	*p*	R^2^-model	*p*-*F*-change model
	With ICV correction					Without ICV correction				
Amygdala	Age	−.416	**.001**	.304	**<.001**	Age	−.447	**.001**	.206	**.002**
						Sex	−.296	**.002**		
Nucleus Accumbens	Age	−.482	**.001**	.372	**<.001**	Age	−.513	**<.001**	.33	**<.001**
						Sex	−.308	**<.001**		
Caudate Nucleus	Age	−.422	**<.001**	.503	**.02**	Age	−.482	**.001**	.201	**.001**
	Sex	.252	.02			Sex	−.322	**.001**		
Hippocampus	Age	−.353	**.007**	.421	**<.001**	Age	−.391	**.004**	.229	**<.001**
						Sex	−.342	**<.001**		
Globus Pallidum	Age	−.51	**<.001**	.364	**<.001**	Age	−.54	**<.001**	.198	**.006**
						Sex	−.262	**.006**		
						Group	−.778	.053		
						Group × age	.82	**.05**		
Putamen	Age	−.612	**<.001**	.496	**<.001**	Age	−.642	**<.001**	.355	**.002**
						Sex	−.272	**.002**		
Thalamus	Age	−.481	**<.001**	.56	**<.001**	Age	−.527	**<.001**	.386	**<.001**
						Sex	−.445	**<.001**		
Cerebellum	Age	−.321	**.005**	.451	**<.001**	Age	−.371	**.003**	.336	**<.001**
						Sex	−.505	**<.001**		
Cerebellum GM	Age	−.284	.02	.366	**<.001**				.06	.113
Total cortical WM				.59	.081	Age	−.302	**.026**	.22	**<.001**
						Sex	−.365	**<.001**		
Total gray	Age	−.462	**<.001**	.732	**<.001**	Age	−.518	**<.001**	.422	**<.001**
						Sex	−.525	**<.001**		
Total Brain (GM + WM)	Age	−.272	**.001**	.698	**<.001**				.052	.164
ICV^a^									.064	.074

Numbers in bold represent significant effects after Holm-Bonferroni correction

Stepwise regression analyses were performed on the whole sample with ICV as correction variable, then group, age, group-by-age interaction term, followed by sex and handedness and IQ as predictors. In the right part of the table, ICV was excluded from the regression analyses

*GM* gray matter, *WM* white matter, *ICV* intracranial volume

^a^Not corrected for ICV volume

Given the strong nature of our age effects and to be able to more easily compare our findings to those reported in the literature, we divided both groups from our sample in a “younger” and “older” group based on their respective median ages for exploratory purposes. This resulted in a: (1) younger group with 26 ASD, mean age = 40.99 (SD = 1.22) and 24 COMs, mean age = 39.99 (1.07); and an older group: (2) 25 ASD, mean age = 62.35 (SD = 1.4), and 17 COMs, mean age = 59.88 (SD = 1.51). There were, as expected, no differences in demographics (*p*’s > .24), except for AQ-scores (*p*’s < .001). In the “older” group, the *l*GI of the insular cortex showed a group-by-age interaction (R^2^ = .27, *p* = .012, β = −2.63, *p* = .025) with the older ASD group showing an age-related decline of *l*GI, but not in COMs. This interaction effect was driven by the *l*GI of the right hemisphere (left: R^2^ = .145, *p* = .064; right: R^2^ = .233, *p* = .04, β = −3.33, *p* = .006). No other significant results were found. Hence, the “younger” adult ASD group did not differ from the “younger” COM group.

### Lateralization Index

Finally, lateralization indices were compared between groups (Table 4). No group differences were found after Bonferroni (15 brain regions with 1–4 brain indices) correction for multiple comparisons *p* < .002. Adopting a liberal threshold (15 brain regions) with *p* < .003, or a Holm-Bonferroni approach did not change our negative laterality findings.

**Table 4:** Lateralization indices for lobar and (sub)cortical brain regions

Brain area	Measure	ASD		COM		Uncorrected *p*	*η*
		Mean	SD	Mean	SD		
Nucleus accumbens	Volume	.015	.076	.030	.066	.34	.01
	Volume						
Amygdala	Volume	−.072	.060	−.050	.056	.053	.039
	Volume						
Caudate nucleus	Volume	−.038	.021	−.036	.020	.667	.002
	Volume						
Cerebellum	Volume	−.004	.012	−.003	.011	.574	.003
	Volume						
Cerebellum GM	Volume	−.002	.018	.002	.018	.28	.012
	Volume						
Hippocampus	Volume	−.012	.027	−.023	.026	.053	.039
	Volume						
Globus pallidum	Volume	−.014	.054	−.027	.053	.254	.014
	Volume						
Putamen	Volume	.017	.026	.008	.039	.182	.019
	Volume						
Thalamus	Volume	.032	.029	.037	.039	.383	.008
	Volume						
ACC	Volume	.021	.056	−.005	.063	.031	.048
	CT	.014	.027	.000	.019	.005	.079
	Surface area	.013	.050	−.001	.064	.213	.016
	*l*GI	−.013	.015	−.008	.020	.221	.016
Frontal	Volume	−.007	.010	−.007	.014	.867	<.001
	CT	−.004	.009	−.003	.008	.517	.007
	Surface area	−.001	.010	−.001	.013	.974	<.001
	*l*GI	.002	.010	.001	.016	.692	.002
Insula	Volume	.003	.041	.004	.033	.958	<.001
	CT	.002	.027	.001	.021	.821	.001
	Surface area	−.004	.057	.001	.043	.742	.001
	*l*GI	.005	.027	.004	.034	.828	<.001
Occipital	Volume	−.005	.035	−.009	.031	.538	.004
	CT	−.001	.016	.001	.014	.576	.003
	Surface area	−.006	.029	−.012	.025	.273	.013
	*l*GI	−.014	.013	−.014	.019	.915	<.001
Parietal	Volume	−.007	.017	−.013	.023	.133	.024
	CT	.007	.009	.007	.010	.842	<.001
	Surface area	−.014	.016	−.019	.028	.161	.021
	*l*GI	−.004	.011	−.007	.016	.288	.012
Temporal	Volume	.013	.017	.018	.015	.137	.023
	CT	−.002	.013	.003	.017	.167	.020
	Surface area	.014	.016	.016	.016	.654	.002
	*l*GI	.007	.015	.002	.023	.147	.022

Lateralization indices. Positive values reflect leftward lateralization, negative values rightward lateralization

Values in bold indicate significant lateralization effects after correcting for multiple comparisons

*GM* gray matter, *ACC* anterior cingulate cortex, *CT* cortical thickness, *lGI* local gyrification index, *η* partial eta squared

## Discussion

Over the past decades, numerous structural MRI studies have investigated ASD-related deficiencies in GM morphology; however, these studies have generally focused on children, adolescents and young adults, and often included only one specific brain measure. Those studies that did focus on adults, focused on early- and mid-adulthood and even often included participants well below adulthood. To our knowledge, this is one of the first reports of a comprehensive assessment of structural brain indices in ASD of adulthood including also those in late adulthood. In contrast to our expectations based on studies with younger participants, no group differences were found in cortical GM volume, thickness, surface area or gyrification. Similarly, subcortical volumes did not differ between groups nor were there any laterality effects. We did, however, replicate some often-observed general age effects (Raz et al. 2010; Walhovd et al. 2005). Yet, no moderating effects of age on the association between GM morphology and diagnosis even were observed suggesting a parallel age-related decline of GM in those with and without ASD.

At first glance our findings may not seem to converge with those reported in the literature showing altered gray matter morphology in various brain regions (Ecker et al. 2012, 2013; Osipowicz et al. 2015; Zielinski et al. 2014). However, our results confirm and extent prior findings from MRI-studies in adults with ASD. Indeed, similar to Raznahan and colleagues (Raznahan et al. 2010) no between group differences in cortical volume and cortical thickness in frontal, parietal and occipital lobes were found. In addition, intracranial, total brain (gray and white matter) and GM volume were similar between groups (Ecker et al. 2013; Lange et al. 2015; McAlonan et al. 2002; Scheel et al. 2011; Toal et al. 2010; Via et al. 2011). Our findings also build upon a recent report from one of the largest anatomical MRI-studies in ASD to date (6–35 years), in which brain anatomical differences between autistic and typically developing individuals were indistinguishable (Haar et al. 2014). Aberrant cortical folding patterns have been reported in children and adolescents (Hardan et al. 2004), and up to young adulthood (Libero et al. 2014; Wallace et al. 2013), but see (Hardan et al. 2004). This may suggest that the marked brain overgrowth in early childhood followed by abnormally slow (and arrest of) growth in late childhood and adolescence is specific for this developmental period (Courchesne et al. 2011), and does not lead to widespread and pervasive consequences in GM in later life. This suggests that atypical development might set off a cascade of events in the early years (including increase of neurons, dendritic growth and synapses, and number and size of glial cells; (Palmen et al. 2004; Schumann and Nordahl 2011)), ultimately resulting in aberrant and dysfunctional communication between neurons which remains stable across the lifespan life. Thus aberrant developmental trajectories and formation of gray matter in early life form the base of ASD-characteristic behavior, and consequently, pursuing GM morphology in late life may not be fruitful. However, caution is warranted for three reasons. First, our exploratory analyses suggested an age-relate declined in *l*GI of the insular cortex in “older” adults with ASD, which was absent in older comparison subjects. It would be of interest to study a group of even older adults to determine whether age-related differences are stronger in older adults with ASD. Secondly, cross-sectional research is not suited to pinpoint dynamic changes and trajectories. Importantly, Lange and colleagues demonstrated no cross-sectional differences in gray matter between ASD and controls, yet their longitudinal approach resulted in atypical age-related trajectories for specific gray matter regions. Therefore, longitudinal research is needed to test these hypotheses. Finally, with the macro-level resolution of current MRI research, we do not have information about possible differences within layers of the GM. Unfortunately findings from post-mortem research do not yet aide our understanding of aging in ASD. Although the majority of post-mortem cases with ASD in the literature are adults, the number of available cases is limited, still less cases above age 30–40, and findings do not converge (Palmen et al. 2004; Schumann and Nordahl 2011).

One could argue that while the sample is unique given the age-range, the sample might have been too small to detect group differences or group-by-age interactions. However, there was enough power to detect the general strong age-related effects and the findings of our exploratory analyses in a small “older age” group hinted towards a steeper age-related decrease in those with ASD as compared to older adults without ASD. Given that there are currently openly available databases including GM morphology measures of adults with ASD, we explored whether our findings remained robust when increasing our sample size by 75 % using independent individuals from the ABIDE database (http://fcon-1000.projects.nitrc.org/indi/abide/) with a similar age-range. We still did not observe any group differences in these additional exploratory brain vertex-wise, lobar and sub-cortical analyses (see supplemental material). Unfortunately, retesting the age-related effects was of no use given that the mean age of the additional included ABIDE adults was only 36 years of age and the oldest participant was just 56 years of age. This shows again that when the ASD research community wants to know what happens at an older age we do need to try recruit more older individuals to participate in adult ASD studies.

However, studying older (intellectually able) adults with ASD comes with various challenges, as for example there are to date no standard procedures to assess ASD at old age. Diagnostic challenges might have resulted in a potential alternative explanation for our lack of between-group differences as the majority of the adults included received their official ASD diagnosis in adulthood. This is not uncommon given that almost all of our subjects were older than 10 years when autism was officially introduced in the diagnostic community [DSM-III (American Psychiatric Association 1980)]. This doesn’t mean that problems during childhood and adolescence were absent, but were probably not yet recognized as autistic behaviors (Geurts and Jansen 2012; James et al. 2006). Studies including younger participants sometimes do report on problems during childhood by using the ADI-R to interview caregivers, however, obviously with the age range of our participants this interview is, unfortunately, not feasible so we followed the Dutch Multidisciplinary Guidelines for ASD Assessment (Kan et al. 2013) and the UK NICE guidelines for identification and assessment of autism in adults (http://www.nice.org.uk/guidance/CG142). While a late diagnosis does not by definition mean that behavioral problems are less severe as compared to those receiving an earlier diagnosis, the late diagnosis might imply that we included a sample with relatively mild ASD symptomatology. Adults with an established ASD diagnosis tend to have lower scores on self-report questionnaires (Bishop and Seltzer 2012). However, our mean AQ-scores match those from a large recent study (Ruzich et al. 2015). With respect to the ADOS, a part of the ASD sample did not meet ADOS-cutoff scores, even though these individuals scored above the clinical cutoff on the AQ. Yet it is known that those with a formal diagnosis of ASD in childhood are not always meeting an ADOS cutoff in adulthood (Anderson et al. 2014; Fein et al. 2013; McGovern and Sigman 2005) and it seems that diagnoses based on a combination of history/caregiver information and clinical observation are significantly more stable over time than results from any single instrument such as the ADOS (Bastiaansen et al. 2011; Jones and Lord 2013; Lord et al. 2006). Given that the AQ and ADOS scores of our sample show little to no differences in terms of current symptom severity with other GM ASD studies (Ecker et al. 2012, 2013; Libero et al. 2014; Scheel et al. 2011; Watanabe et al. 2014), and the fact that all participants were patients from mental health clinics and that the majority was using psychotropic medication, we feel that our sample is with respect to severity highly similar to earlier adulthood studies. Unfortunately a large number of recent brain imaging studies did neither report average scores on ADOS or AQ (Hardan et al. 2004; McAlonan et al. 2002; Raznahan et al. 2010; Rojas et al. 2006; Toal et al. 2010), nor report the age of diagnosis, which hampers direct comparisons. Nonetheless for future research it would be of interest to compare older individuals with a childhood diagnosis directly to those with a diagnosis in adulthood.

## Conclusions

Taken together, the lack of significant anatomical differences between intellectually able individuals with ASD and COMs suggests that ASD in mid and late adulthood is not (strongly) related to GM morphology. These results remained after inclusion of independent age-matched participants from the international ABIDE database and extent prior findings of suggestive GM normalization in late adolescence and early adulthood. Our findings do not indicate a premature aging pattern in ASD, which seems to counter the suggestion of an elevated risk of dementia in the ASD-population (Mukaetova-Ladinska et al. 2012). Studies including even older participants replicating the current findings are needed to determine whether aging in those with ASD is indeed no double jeopardy.

## Electronic supplementary material

Below is the link to the electronic supplementary material.

Fig. S1 Supplementary material 1 (TIFF 9108 kb)

Table S1 Demographic variables ABIDE data set (DOCX 86 kb)

Table S2 Lobar regression analyses for all morphometric measures ADOS-only (DOCX 98 kb)

Table S3 Lobar regression analyses for all morphometric measures including ABIDE sample (total N=177) (DOCX 103 kb)

Table S4 Volumes of cortical and subcortical brain structures ADOS-only (DOCX 65 kb)

Table S5 Volumes of cortical and subcortical brain structures including ABIDE sample (total N=177) (DOCX 99 kb)

Doc S Supplemental Material ABIDE. Description, Methods and Results (DOCX 94 kb)
