# Supplementary figures and images for: Gray Matter Characteristics in Mid and Old Aged Adults with ASD
Source: J Autism Dev Disord. 2016 May 13;46:2666–78. doi: 10.1007/s10803-016-2810-9 (PMC4938851; doi:10.1007/s10803-016-2810-9)

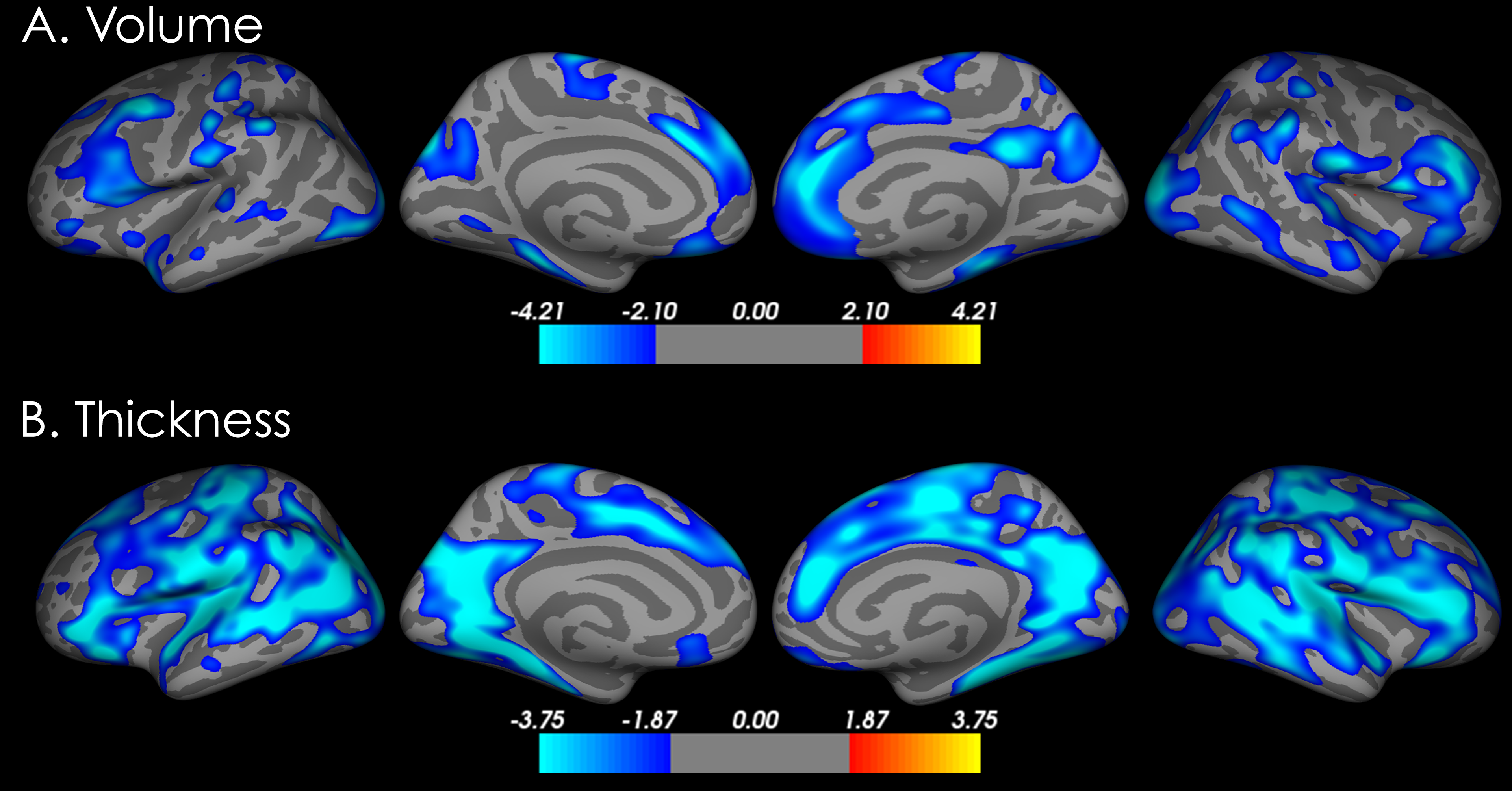

Supplement: Supplementary file 1 — Supplementary material 1 (TIFF 9108 kb) [file 10803_2016_2810_MOESM1_ESM.tif]
