# Supplementary material for: Gray Matter Characteristics in Mid and Old Aged Adults with ASD
Source: J Autism Dev Disord. 2016 May 13;46:2666–78. doi: 10.1007/s10803-016-2810-9 (PMC4938851; doi:10.1007/s10803-016-2810-9)
Supplement: Supplementary file 2 — Demographic variables ABIDE data set (DOCX 86 kb) [file 10803_2016_2810_MOESM2_ESM.docx]

| **Description^a^** | **ABIDE ASD** | **ABIDE COM** | **Statistics^b^** |
| --- | --- | --- | --- |
|  | N=34 | N=43 |  |
| #Males (%) | 30 (88%) | 39 (91%) | **ASD-ABIDE ASD *χ^2^*=4.36, p=.037**  **COM-ABIDE COM**  ***χ^2^*=8.38, p=.004** |
| Age (SD)[range] | 36.12 (6.27)  [30-55.4] | 36.46 (5.89)  [30-56.2] | **ASD-ABIDE ASD *F=43.1, p<.001***  **COM-ABIDE COM**  ***F=46.5, p<.001*** |
| IQ (SD)[range] | 109.93 (18.06)  [65-134]**^c^** | 112.12 (9.45)  [95-129]**^d^** | **ASD-ABIDE ASD** *F=2.53, p=.116*  **COM-ABIDE COM**  *F=.03, p=.863* |
| Handedness |  |  | **ASD-ABIDE ASD *χ^2^*=24.24, p<.001**  **COM-ABIDE COM**  ***χ^2^*=17.34, p<.001** |
| Left | 20 | 19 |  |
| Right | 12 | 24 |  |
| Ambidexter | 2 | 0 |  |
| ADOS Total^h^ | 12.3 (3.46) | *N.A.* | **ASD-ABIDE ASD *F=31.74, p<.001*** |
| AQ Total | 35.89 (5.16)  [26-42]**^e^** | *N.A.* | **ASD-ABIDE ASD** *F=.02, p=.887* |
| Medication N (%) | 7, *17 missing* | 0, *11 missing* | **ASD-ABIDE ASD**  *χ^2^*=38.35, p<.001 |
| Antidepressants | 2 | 0 |  |
| Antipsychotics | 1 | 0 |  |
| Sedatives | 1 | 0 |  |
| Stimulants | 2 | 0 |  |
| Antiepileptics | 0 | 0 |  |
| Antiparkinson | 0 | 0 |  |
| Migraine | 0 | 0 |  |
| Non-psychotropic medication | 1 | 0 |  |
| Note. Numbers in bold reflect significant between group differences.  ^a^ Information on MMSE, Level of educational attainment, Age at first diagnosis, ADOS subscales were not available in ABIDE database.  ^b^  Comparisons based on different cohorts.  ^c^ Available for 27 subjects; based on various measures including WAIS, WISC, WASI * WST.  ^d^ Available for 33 subjects; based on various measures including WAIS, WISC, WASI * WST.  ^e^ Available for 9 subjects.  Abbreviations: ASD, autism spectrum disorder; COM, comparison group; ADOS, Autism Diagnostic Observation Schedule; AQ, Autism-Spectrum quotient; MMSE, Mini-mental state examination; N.A., not applicable. | | | |
