# Supplementary material for: Gray Matter Characteristics in Mid and Old Aged Adults with ASD
Source: J Autism Dev Disord. 2016 May 13;46:2666–78. doi: 10.1007/s10803-016-2810-9 (PMC4938851; doi:10.1007/s10803-016-2810-9)
Supplement: Supplementary file 3 — Lobar regression analyses for all morphometric measures ADOS-only (DOCX 98 kb) [file 10803_2016_2810_MOESM3_ESM.docx]

**S2 Table. Lobar regression analyses for all morphometric measures ADOS-only.**

| A | **Volumes** |  |  |  |  |  |
| --- | --- | --- | --- | --- | --- | --- |
|  | **Lobes** | **Description** | **ß** | ***p*** | **R^2^-model** | ***p*-F-change** |
|  | **ACC** | age | -.342 | **.001** | .53 | **.001** |
|  | **Frontal** | age | -.348 | **<.001** | .627 | **<.001** |
|  | **Insula** | group | .64 | .048 | .598 | **.006** |
|  |  | group-x-age | -.626 | *.055* |  |  |
|  | **Occipital** | age | -.409 | **<.001** | .611 | **<.001** |
|  | **Parietal** | age | -.484 | **<.001** | .73 | **<.001** |
|  | **Temporal** | age | -.363 | **<.001** | .511 | **<.001** |
| B | **Thickness** |  |  |  |  |  |
|  | **Lobes** | **Description** | **ß** | ***p*** | **R^2^-model** | ***p*-F-change** |
|  | **ACC** | age | -.555 | **<.001** | .347 | **.02** |
|  |  | sex | .22 | .02 |  |  |
|  | **Frontal** | age | -.479 | **<.001** | .267 | **<.001** |
|  | **Insula** | age | -.434 | **.001** | .201 | **.001** |
|  | **Occipital** | age | -.599 | **<.001** | .38 | **<.001** |
|  | **Parietal** | age | -.68 | **<.001** | .467 | **<.001** |
|  | **Temporal** | age | -.475 | **<.001** | .267 | **<.001** |
| C | **Surface area** |  |  |  |  |  |
|  | **Lobes** |  |  |  | **R^2^-model** | ***p*-F-change** |
|  | **ACC** |  |  |  | .03 | .494 |
|  | **Frontal** |  |  |  | .046 | .301 |
|  | **Insula** |  |  |  | .086 | .902 |
|  | **Occipital** |  |  |  | .036 | .409 |
|  | **Parietal** |  |  |  | .035 | .328 |
|  | **Temporal** |  |  |  | .057 | .204 |
| D | ***l*GI** |  |  |  |  |  |
|  | **Lobes** | **Description** | **ß** | ***p*** | **R^2^-model** | ***p*-F-change** |
|  | **ACC** |  |  |  | .033 | .445 |
|  | **Frontal** | age | -.351 | **.008** | .217 | **.021** |
|  |  | sex | -.238 | .021 |  |  |
|  |  | sex-x-age^a^ | -.214 | .494 |  |  |
|  | **Insula** |  |  |  | .079 | .091 |
|  | **Occipital** |  |  |  | .036 | .407 |
|  | **Parietal** | age | -.284 | .035 | .174 | **.023** |
|  |  | sex | -.241 | .023 |  |  |
|  |  | sex-x-age^a^ | .175 | .586 |  |  |
|  | **Temporal** |  |  |  | .07 | .127 |
| Note. ADOS only: group comparisons with ASD group above cut-off score ADOS (>7).  Numbers in bold represent significant effects after Holm-Bonferroni correction.  ^a^ explorative analyses  Abbreviations: ACC, anterior cingulate cortex; *l*GI, local gyrification index. | | | | | | |
