# Supplementary material for: Gray Matter Characteristics in Mid and Old Aged Adults with ASD
Source: J Autism Dev Disord. 2016 May 13;46:2666–78. doi: 10.1007/s10803-016-2810-9 (PMC4938851; doi:10.1007/s10803-016-2810-9)
Supplement: Supplementary file 4 — Lobar regression analyses for all morphometric measures including ABIDE sample (total N=177) (DOCX 103 kb) [file 10803_2016_2810_MOESM4_ESM.docx]

**S3 Table. Lobar regression analyses for all morphometric measures including ABIDE sample (total N=177).**

| A | **Volumes^a^** |  |  |  |  |  |
| --- | --- | --- | --- | --- | --- | --- |
|  | **Lobes** | **Description** | **ß** | ***p*** | **R^2^-model** | ***p*-F-change** |
|  | **ACC** | age | -.194 | **.022** | .455 | **.003** |
|  | **Frontal** | age | -.300 | **<.001** | 674 | **<.001** |
|  | **Insula** |  |  |  | .600 | **.013** |
|  | **Occipital** | age | -.257 | **.005** | .372 | **.001** |
|  | **Parietal** | age | -.241 | **.001** | .602 | **<.001** |
|  |  | sex | .128 | **.042** |  |  |
|  | **Temporal** | age | -.312 | **<.001** | .633 | **<.001** |
| B | **Thickness** |  |  |  |  |  |
|  | **Lobes** | **Description** | **ß** | ***p*** | **R^2^-model** | ***p*-F-change** |
|  | **ACC** |  |  |  | .031 | .136 |
|  | **Frontal** | age | -.362 | **.001** | .119 | **<.001** |
|  | **Insula** |  |  |  | .041 | .066 |
|  | **Occipital** |  |  |  | .022 | .283 |
|  | **Parietal** | age | -.379 | **<.001** | .143 | **<.001** |
|  | **Temporal** | age | -.453 | **<.001** | .201 | **<.001** |
| C | **Surface area** |  |  |  |  |  |
|  | **Lobes** |  |  |  | **R^2^-model** | ***p*-F-change** |
|  | **ACC** | sex | -.383 | **<.001** | .229 | **<.001** |
|  | **Frontal** | sex | -.451 | **<.001** | .332 | **<.001** |
|  | **Insula** | sex | -.434 | **<.001** | .231 | **<.001** |
|  | **Occipital** | sex | -.435 | **<.001** | .295 | **<.001** |
|  | **Parietal** | sex | -.439 | **<.001** | .283 | **<.001** |
|  | **Temporal** | sex | -.441 | **<.001** | .309 | **<.001** |
| D | ***l*GI** |  |  |  |  |  |
|  | **Lobes** | **Description** | **ß** | ***p*** | **R^2^-model** | ***p*-F-change** |
|  | **ACC** | age | -.324 | **.001** | .217 | **.002** |
|  |  | sex | -.218 | **.002** |  |  |
|  | **Frontal** | age | -.472 | **<.001** | .286 | **.003** |
|  |  | sex | -.196 | **.003** |  |  |
|  | **Insula** | age | -.376 | **<.001** | .241 | **<.001** |
|  |  | sex | -.266 | **<.001** |  |  |
|  | **Occipital** | age | -.341 | **<.001** | .227 | **.003** |
|  |  | sex | -.206 | **.003** |  |  |
|  | **Parietal** | age | -.445 | **<.001** | .288 | **.001** |
|  |  | sex | -.225 | **.001** |  |  |
|  | **Temporal** | age | -.379 | **<.001** | .228 | **<.001** |
|  |  | sex | -.243 | **<.001** |  |  |
| Note. Numbers in bold represent significant effects after Holm-Bonferroni correction.  ^a^ With ICV correction  Abbreviations: ACC, anterior cingulate cortex; *l*GI, local gyrification index. | | | | | | |
