# Supplementary material for: Gray Matter Characteristics in Mid and Old Aged Adults with ASD
Source: J Autism Dev Disord. 2016 May 13;46:2666–78. doi: 10.1007/s10803-016-2810-9 (PMC4938851; doi:10.1007/s10803-016-2810-9)
Supplement: Supplementary file 5 — Volumes of cortical and subcortical brain structures ADOS-only (DOCX 65 kb) [file 10803_2016_2810_MOESM5_ESM.docx]

**S4 Table. Volumes of cortical and subcortical brain structures ADOS-only.**

| **(sub)cortical brain volumes** | | | | | |
| --- | --- | --- | --- | --- | --- |
| **Brain area** | **Description** | **ß** | ***p*** | **R^2^-model** | ***p*-*F*-change model** |
| **Amygdala** | age | -.39 | **.002** | .28 | **.004** |
| **Nucleus Accumbens** | age | -.479 | **.001** | .391 | **<.001** |
| **Caudate Nucleus** | age | -.4 | **<.001** | .526 | **<.001** |
| **Caudate Nucleus** | sex | .27 | .022 |  |  |
| **Hippocampus** | age | -.324 | **.004** | .432 | **<.001** |
| **Globus Pallidum** | age | -.47 | **<.001** | -.387 | **<.001** |
| **Putamen** | age | -.573 | **<.001** | .474 | **<.001** |
| **Thalamus** | age | -.449 | **<.001** | .592 | **<.001** |
| **Cerebellum** | age | -.295 | **.005** | .496 | **<.001** |
| **Cerebellum GM** | age | -.319 | **.005** | .411 | **<.001** |
| **Total gray (GM+WM)** | age | -.421 | **<.001** | .732 | **<.001** |
| **Total Brain** | age | -.257 | **.002** | .704 | **<.001** |
| **ICV** |  |  |  | .014 | .781 |
| Note. Numbers in bold represent significant effects after Holm-Bonferroni correction. ADOS only: group comparisons with ASD group above cut-off score ADOS (>7).  Abbreviations: GM, gray matter; ICV, intracranial volume. | | | | | |
