# Supplementary material for: Gray Matter Characteristics in Mid and Old Aged Adults with ASD
Source: J Autism Dev Disord. 2016 May 13;46:2666–78. doi: 10.1007/s10803-016-2810-9 (PMC4938851; doi:10.1007/s10803-016-2810-9)
Supplement: Supplementary file 6 — Volumes of cortical and subcortical brain structures including ABIDE sample (total N=177) (DOCX 99 kb) [file 10803_2016_2810_MOESM6_ESM.docx]

**S5 Table. Volumes of cortical and subcortical brain structures including ABIDE sample (total N=177).**

| **Brain area** | **Description** | **ß** | ***p*** | **R^2^-model** | ***p*-*F*-change**  **model** | **Description** | **ß** | ***p*** | **R^2^-model** | ***p*-*F*-change model** |
| --- | --- | --- | --- | --- | --- | --- | --- | --- | --- | --- |
|  | **with ICV correction** | | | | | **without ICV correction** | | | |  |
| **Amygdala** |  |  |  | .215 | **.003** | age | -.248 | **.016** | .161 | **<.001** |
|  |  |  |  |  |  | sex | -.253 | **<.001** |  |  |
| **Nucleus Accumbens** |  |  |  | .002 | .536 |  |  |  | .016 | .426 |
| **Caudate Nucleus** | age | -.227 | **.013** | .37 | **.034** | age | -.368 | **<.001** | .155 | **<.001** |
|  |  |  |  |  |  | sex | -.259 | **<.001** |  |  |
| **Hippocampus** | group-x-age | -.473 | .05 | .388 | **<.001** | age | -.277 | **.005** | .238 | **<.001** |
|  |  |  |  |  |  | sex | -.311 | **<.001** |  |  |
| **Globus Pallidum** | age | -.242 | **.016** | .23 | **.002** | age | -.326 | **.002** | .169 | **<.001** |
|  |  |  |  |  |  | sex | -.252 | **<.001** |  |  |
| **Putamen** | age | -.283 | **.005** | .228 | **<.001** | age | -.355 | **.001** | .175 | **.017** |
|  |  |  |  |  |  | sex | -.168 | **.017** |  |  |
| **Thalamus** | age | -.359 | **<.001** | .682 | **<.001** | age | -.510 | **<.001** | .453 | **<.001** |
|  |  |  |  |  |  | sex | -.362 | **<.001** |  |  |
| **Cerebellum** | age | -.158 | .061 | .458 | **<.001** | age | -.301 | **.002** | .251 | **<.001** |
|  |  |  |  |  |  | sex | -.342 | **<.001** |  |  |
| **Cerebellum GM** |  |  |  | .327 | **.019** | age | -.248 | **.017** | .154 | **<.001** |
|  |  |  |  |  |  | sex | -.276 | **<.001** |  |  |
| **Total cortical WM** |  |  |  | .529 | **<.001** |  |  |  | .021 | .304 |
|  |  |  |  |  |  |  |  |  |  |  |
| **Total gray** | age | -.306 | **<.001** | .743 | **<.001** | age | -.487 | **<.001** | .407 | **<.001** |
|  |  |  |  |  |  | sex | -.409 | **<.001** |  |  |
| **Total Brain (GM+WM)** | age | -.125 | **.039** | .721 | **<.001** | age | -.317 | **<.001** | .364 | **<.001** |
|  |  |  |  |  |  | sex | -.492 | **<.001** |  |  |
| **ICV*** |  |  |  |  |  | age | -.241 | .004 | .441 | <.001 |
|  |  |  |  |  | sex | -.629 | <.001 |  |  |  |
| Note. Numbers in bold represent significant effects after Holm-Bonferroni correction.  Stepwise regression analyses were performed on the whole sample with ICV as correction variable, then group, age, group-by-age interaction term, followed by sex and handedness and IQ as predictors. In the right part of the table, ICV was excluded from the regression analyses. * Not corrected for ICV volume. Abbreviations: GM, gray matter; WM, white matter; ICV, intracranial volume. | | | | | | | | | | |
