# Supplementary material for: Gray Matter Characteristics in Mid and Old Aged Adults with ASD
Source: J Autism Dev Disord. 2016 May 13;46:2666–78. doi: 10.1007/s10803-016-2810-9 (PMC4938851; doi:10.1007/s10803-016-2810-9)
Supplement: Supplementary file 7 — Supplemental Material ABIDE. Description, Methods and Results (DOCX 94 kb) [file 10803_2016_2810_MOESM7_ESM.docx]

# Supplemental Material ABIDE

# Methods

For exploratory purposes, an additional patient and control cohort was included from the open-access Autism Brain Imaging Exchange (ABIDE; <http://fcon_1000.projects.nitrc.org/indi/abide/>) dataset to address across-site reproducibility and increase sample size [[50](#_ENREF_50)]. With our focus on mid and late adulthood, only adults aged 30 years and beyond were included. This resulted in 91 individuals (48 ASD, 43 COM (comparison group)) from eight different sites. Due to differences in criteria for diagnosing patients with autism across sites, we applied our current inclusion criteria, i.e. ADOS≥7 and those not scoring above this cut off did score above the AQ cutoff (≥26), resulting in a final sample of 34 individuals with ASD and 43 COMs (**Supplemental** **Table 1**).

For the ABIDE dataset (34 ASD/43 COM), we computed the same measures using the same procedures as described for our main analyses. We repeated each analysis (whole brain vertex-wise and ROI) combining the ABIDE sample with our own (N_total_= 85 ASD/92 COM).

# Results

## Whole brain vertex-wise analyses

Inclusion of the ABIDE subjects, and thus increasing sample size, resulted in replicating our own findings. Due to the skewed sex distribution and taking into account that most other studies are limited to males, we ran analyses taking sex into account and limiting our sample to males only. However, none of these sub-analyses revealed differences between groups on a whole brain level.

## Lobar analyses

After inclusion of the ABIDE subjects, still no group differences were found. There were, however, some changes in age- and sex-effects due to the skewed distribution of those variables in the ABIDE sample compared to our own sample (**S3 Table**).

## Subcortical analyses

Similar to the lobar analyses, no group differences emerged after inclusion of the ABIDE sample (**S5 Table**).

## Lateralization Index

Inclusion of the ABIDE sample resulted in two small laterality effects on putamen volume (F=5.49, p=.02, η=.03) and cingulate thickness (F=7.32, p=.008, η=.04), such that left was larger than right.
